# Foreign Bodies in the Stomach

**Published:** 1848

**Authors:** 


### Article VII.—*Foreign Bodies in the Stomach.*

M. Gaide reported to the Medico-Practical Society of Paris, the case of a young man who had gotten a fish-bone in his throat, and who in attempting to remove it with a fork, this latter slipped into the pharynx. Consulting an officer of health, he, instead of removing the fork, advised some sweet oil to the throat. In short, the patient swallowed the fork, which still remains in his stomach. His health has not suffered much, he only felt slight pain each day at the period of digestion. M. G. mentions another case in which he made a post-mortem examinationof a patient who had often told him he had swallowed a fork ten years before his death. He found, in fact, the fork in his stomach, which was placed longitudinally to its great curvature, the four prongs being enveloped and fixed in voluminous fleshy projections. In connection with this subject, the American reader must be told that the forks of France are made of silver and are four-pronged.—*Southern Med. and Surg. Jour.*

